# Local drug delivery in the treatment of furcation defects in periodontitis: a systematic review

**DOI:** 10.1007/s00784-023-04871-0

**Published:** 2023-02-02

**Authors:** Georgios S. Chatzopoulos, Vasiliki P. Koidou, Lazaros Tsalikis

**Affiliations:** 1 Department of Preventive Dentistry, Periodontology and Implant Biology, School of Dentistry, Aristotle University of Thessaloniki, Thessaloniki, Greece; 2 Division of Periodontology, Department of Developmental and Surgical Sciences, School of Dentistry, University of Minnesota, 515 Delaware Street SE, Minneapolis, MN 55455 USA; 3 Centre for Oral Immunobiology and Regenerative Medicine and Centre for Oral Clinical Research, Institute of Dentistry, Queen Mary University London (QMUL), London, UK

**Keywords:** Periodontitis; Host-modulating agents, Local drug delivery systems, Non-surgical periodontal therapy, Adjunctive therapy, Systematic review

## Abstract

**Objectives:**

To evaluate the effect of subgingival administration of various antimicrobials and host-modulating agents in furcation defects as an adjunct to scaling and root planing (SRP) compared to SRP alone or combined with placebo.

**Methods:**

A systematic review was carried out using MEDLINE-PubMed, Embase, and Scopus for articles up to October 2022 in addition to hand searches. All longitudinal studies that evaluated the effect of subgingival application of antimicrobial and host-modulating agents in furcation defects as adjuncts to SRP compared to SRP alone or SRP + placebo with at least 3 months of follow-up were eligible for inclusion.

**Results:**

A total of eight studies were included. Superior clinical treatment outcomes were shown when alendronate, rosuvastatin, boric acid, simvastatin, and tetracycline (only at 3 months) were utilized in furcation defects in conjunction with SRP alone or SRP + placebo. Significant improvement was reported in radiographic bone defect depth and defect depth reduction when SRP was supplemented with alendronate, rosuvastatin, boric acid, and simvastatin.

**Conclusions:**

Within the limitations of this review, the adjunctive subgingival administration of medications and host-modulating agents in furcation defects may confer additional clinical and radiographic benefits than non-surgical periodontal treatment alone. Future investigations are needed to confirm their long-term effectiveness.

**Clinical relevance:**

Local host modulators and antimicrobials may be used supplementary to enhance the clinical and radiographic treatment outcomes of conventional periodontal therapy in furcation defects.

## Introduction

Periodontitis is a multifactorial inflammatory disease affecting the tissues surrounding the teeth and it is characterized by loss of connective tissue attachment and alveolar bone [1]. Untreated periodontitis may ultimately lead to tooth loss, which may significantly affect individuals’ function, esthetics, and physiology [2, 3]. Periodontitis is induced by a dysbiotic dental biofilm and it is a result of an imbalance between the microbial biofilm and the host response leading to progressive destruction of the periodontium [4, 5]. According to the Global Burden of Disease Study, it has been estimated that severe periodontitis has affected 1.1 billion people worldwide thereby posing a significant economic burden in nations [6, 7].

Non-surgical periodontal therapy through scaling and root planing (SRP) is currently considered the gold standard for the treatment of periodontitis and aims at reducing soft tissue inflammation by removing both soft and hard dental biofilm deposits [8]. However, this treatment approach has limitations and may not be beneficial universally [9]. Recurrent disease, deep periodontal pockets, difficulty of access, and irregular root surfaces complicate the removal of biofilm deposits and may pose a need for adjunctive therapies [9]. In addition, individuals susceptible to periodontitis may require additional interventions to overcome the ecological challenges [10, 11].

When teeth with furcation defects are considered, it is well established that they exhibit a higher tooth loss rate than those without such anatomical defects [12]. The anatomical characteristics, the limited accessibility, and the complexity of the healing of the furcation defects are the main challenges associated with such teeth [13, 14]. The complete removal of subgingival biofilm and calculus in furcations may be difficult when root debridement is solely performed, and therefore further treatment should be implemented [15]. Non-responding sites may be treated with repeated subgingival instrumentation with or without adjunctive therapies or surgically following the protocols of access flap, resective, or regenerative periodontal surgery [15].

Adjuncts to non-surgical periodontal treatment have been proposed to enhance the clinical and microbiological outcomes in periodontitis patients, including local delivery of antimicrobials, antibiotics (local or systemic use), and host modulation agents [10, 15]. The use of local antimicrobials can lead to significant improvements in periodontal clinical outcomes, without side effects, when compared to subgingival debridement alone [16, 17]. In addition, the use of host modulators has shown beneficial results [18]. The application of local adjuncts could possibly reduce the risk of adverse events and development of antibiotic resistance from systemic antimicrobials [19]. Antimicrobials encompass a group of agents that aim to reduce the possibility of infection and sepsis and target bacteria, fungi, viruses, and protozoa non-specifically (broad spectrum activity). Antibiotics may lead to microbial resistance and target specific bacteria only (narrow spectrum activity). They can either inhibit their growth (bacteriostatic) or kill them (bactericidal) [20].

The aim of periodontal treatment is to arrest disease progression by reducing the bacterial load and to reduce the risk of tooth loss, as well as to prevent disease recurrence, even in restricted access areas, such as furcation defects. Local adjunctive periodontal therapy may be utilized in such areas to overcome these limitations. No systematic review has evaluated the available evidence regarding all available agents on the effect of local adjunctive non-surgical periodontal treatments in furcation areas. In this systematic review, both short- and long-term clinical and radiographic findings are assessed following in previously untreated periodontitis patients as well as in patients with recurrent periodontitis during supportive periodontal care. Findings from this systematic review will enable clinicians to exercise evidence-based decision-making for the management of periodontitis patients with furcation defects. Therefore, the aim of this systematic review was to evaluate the effect of subgingival administration of various drugs in furcation defects as an adjunct to SRP compared to SRP alone or combined with placebo.

## Material and methods

The present systematic review followed a detailed protocol according to the PRISMA (Preferred Reporting Items Systematic review and Meta-Analyses) guidelines [21]. The protocol for this systematic review was registered in PROSPERO (International Prospective Register of Systematic Reviews) under the ID CRD42022352057.

### PICO question

The formulated PICO (Population, Intervention, Comparison, and Outcome) question was: “In systemically healthy adult patients diagnosed with periodontitis, does subgingival application of medications in furcation defects as an adjunct to non-surgical periodontal therapy (SRP) improve clinical and radiographic periodontal parameters, when compared to non-surgical periodontal therapy alone or combined with placebo?”.

- Population: systemically healthy adult patients diagnosed with periodontitis who exhibit at least one furcation defect. Previously untreated periodontitis patients and patients with recurrent periodontitis during supportive periodontal care were included.
- Intervention: scaling and root planing (SRP) in combination with local application of medications including antimicrobials, antiseptics, host modulators, and biologics in grade II (osseous destruction into the furcation area but not extending to the opposite site) furcation defects.
- Comparison: scaling and root planing (SRP) alone or SRP + placebo.
- Outcome: clinical and radiographic changes following periodontal treatment. The primary outcome of the study was the probing pocket depth (PPD) reduction. Secondary outcomes were the following: relative vertical clinical attachment loss (RVAL), relative horizontal clinical attachment loss (RHAL), clinical attachment level (CAL) gain, bleeding on probing reduction, bone defect fill, and furcation closure as well as adverse events.

### Eligibility criteria

All longitudinal comparative studies with at least 3 months of follow-up were eligible for inclusion—randomized and non-randomized clinical trials that evaluated the effect of subgingival application of drugs in furcation defects as adjuncts to SRP compared to SRP alone or SRP + placebo. Studies performing non-surgical periodontal treatment were only included, while surgical studies were excluded. Included studies reported at least one of the aforementioned outcomes. No language limitations were applied. The exclusion criteria were as follows: (1) abstracts of posters or presentations; (2) studies not including furcation defects; (3) studies including surgical periodontal treatment; (4) studies that did not report treatment outcomes of interest for the present review; (5) studies including less than 3 months of follow-up; (6) case reports; (7) case series.

### Search and screening strategy

Electronic and manual literature searches were conducted by two independent reviewers (G. S. C, V. P. K) in databases including MEDLINE-PubMed, Embase, and Scopus for articles up to October 2022, without language restriction. In addition, a manual search of the reference lists of the included studies and of relevant journals including the Journal of Dental Research, Journal of Clinical Periodontology, Journal of Periodontal Research, and Journal of Periodontology for publications after 2010 were performed. Grey literature was searched on clinicaltrials.gov to identify potential ongoing or completed RCTs. The two reviewers assessed the eligibility of the studies for inclusion in this systematic review independently based on their title and abstract (1st round) and full text (2nd round). Publications that did not meet the inclusion criteria were excluded. Any disagreement was resolved with discussion. In case of disagreement, a third person (L. T.) was able to advise. The level of agreement between the two reviewers was calculated using kappa statistics.

A series of search terms combined with Boolean operators “AND” and “OR” was used. The following search string was developed: (Local application OR topical application OR local drug delivery) AND (furc* OR molar* OR multirooted* OR multi-rooted* OR radicular).

### Data extraction

The characteristics of the included publications were extracted by two reviewers (G. S. C and V. P. K.) independently and any discrepancy was resolved with discussion. Among the characteristics extracted were as follows: (1) general information including authors, title, year and journal of publication, country of origin, study setting, study design, length of follow-up, number of clinicians/evaluators; (2) patient characteristics including number of subjects in each group and smoking status; (3) treatment characteristics including type of intervention and controls, number of treated sites, type of teeth included in the study; (4) outcomes including clinical and radiographic parameters, as well as reported adverse events.

Meta-analysis of the data reported in the included studies was planned to quantitatively assess the effect of the subgingival application of medications/agents in furcation defects as adjuncts to SRP based on a specific protocol. Baseline clinical and radiographic characteristics were summarized as weighted mean differences and 95% confidence intervals. For each parameter, weighted treatment effects (pre-treatment–post-treatment) were to be calculated and expressed as weighted mean differences and standard deviations. Standard pairwise meta-analyses of direct comparisons (test versus control groups) were to be performed using a random-effect model, and results were to be expressed as mean differences and relative 95% confidence intervals. The results of the meta-analysis were to be summarized with forest plots. Heterogeneity was to be assessed by using the *χ*^2^-based *Q*-statistic method and *I*^2^ measurement. A *p* value of 0.05 was set as the level of significance. If the necessary data were available, subgroup analyses were to be done to explore differences in treatment outcomes between different medications/agents, smokers/non-smokers, and time of the intervention (initial treatment or retreatment). Whenever applicable, subgroup analysis would be performed based on the duration of follow-up and type of disease.

### Quality assessment

The risk of bias in the included publications was assessed by two reviewers (G. S. C, V. P. K.) independently and in duplicate. Any discrepancy was resolved with discussion. The quality of the selected randomized clinical trials (RCTs) was assessed according to the revised RoB 2.0 tool (revised tool for assessment of the risk of bias in randomized trials) [22]. In particular, the selected publications were assessed for the following parameters: bias arising from the randomization process, bias due to deviations from intended interventions, bias due to missing outcome data, bias in measurement of the outcome, and bias in selection of the reported result. If however, non-randomized studies were also included after the screening, the Methodological Index For Non-randomized Studies (MINORS) tool was to be employed [23].

## Results

**Fig. 1:**
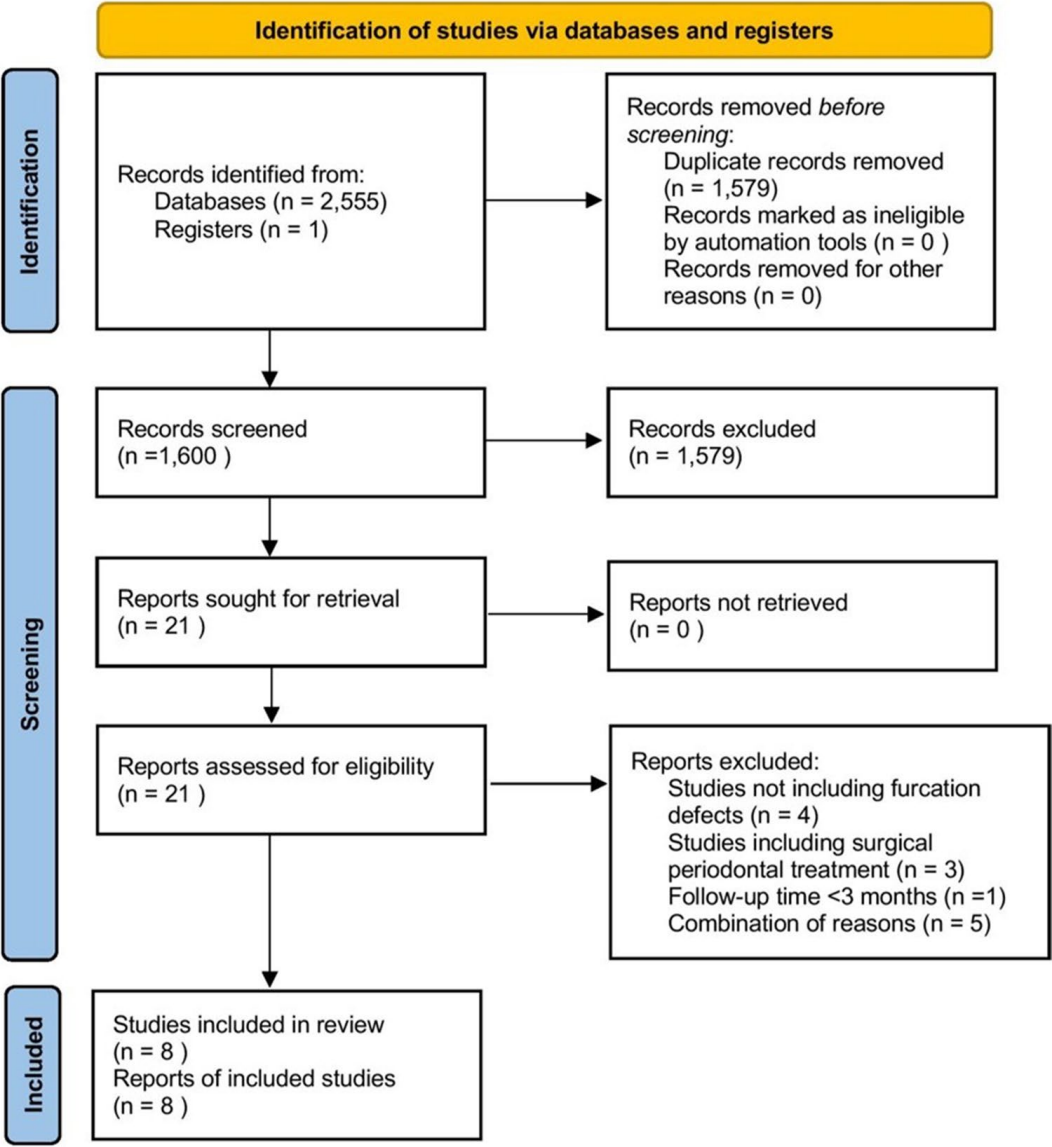
PRISMA flow diagram of the study selection process

The PRISMA flow diagram of the study selection process is shown in Fig. 1. The initial search yielded 2555 articles (Pubmed-MEDLINE: 1298; EMBASE: 1195; and SCOPUS: 62). One additional title was identified through hand search for a total of 1600 records after duplicates removal. Following the first round of screening, a total of 1579 titles were excluded and 21 articles were assessed for eligibility by the reviewers (G. S. C., V. P. K.). Following a full-text review, eight articles were deemed eligible and included in the qualitative synthesis of this systematic review. The kappa score for the inter-reviewer agreement was 0.95 in the first round and 1.0 in the second round.

### Study characteristics

The characteristics of the studies included in the qualitative synthesis are shown in Table 1. All included studies [24–31] were RCTs and were published between 1998 [31] and 2018 [24]. Seven investigations [24–30] were performed in university dental clinics, while one study was a multicenter private practice–based clinical trial [31]. The included population ranged from 32 [29] to 123 [31] and five of the studies included only non-smokers [24–28]. Various adjuncts were utilized including alendronate in two studies [24, 27], doxycycline in two studies [29, 30], rosuvastatin [25], boric acid [26], simvastatin [28], and tetracycline [31] in one study. The majority of the included investigations tested different adjunct treatments during periodontal maintenance [26–31], while the remaining studies [24, 25] applied the adjunctive treatment during the initial active periodontal therapy. Mandibular molars with grade II furcation involvement were primarily evaluated [24–28, 31], whereas two studies included maxillary and mandibular molars with grades I, II, and III furcation defects [29, 30]. The diagnosis used for inclusion was similar among the studies and included PPD of at least 5 mm and furcation involvement. Single treatment providers were used in seven studies [24–30], while the remaining investigation included multiple providers, due to its study design [31]. Similarly, single and blinded examiners were utilized to periodontally assess the included patients in six studies [24–28, 30]. The follow-up time was up to 12 months post-treatment [24, 27, 30]. Two studies included previously untreated periodontitis patients [24, 25], while the rest of the studies used adjunctive drugs in recurrent periodontitis cases in supportive periodontal care [26–31]. The majority of the included studies [24–28] did not report any source of funding for the study, while the remaining three [29–31] received research funds from industry and private companies.

**Table 1:** Characteristics of the individual studies included in the qualitative synthesis

Study	Year published	Country	Study design	Setting	Patients (test/control)	Sites (test/control)	Groups A. Test B. Control	Smokers	Type of furcation/tooth	Periodontal diagnosis for inclusion	Clinician(s)	Evaluator(s)	Follow-up	Examined parameters	Phase of periodontal treatment
Ipshita et al. [24]	2018	India	RCT	University	90 (30, 30, 30)	90 (30, 30, 30)	A.1. SRP + 1% aledronate A.2. SRP + aloe vera B. SRP + placebo	None	Mandibular class II furcations	PD > = 5 mm and horizontal PD > = 3 mm	Single	Single, calibrated, blinded	6 and 12 months	mSBI, PI, PPD, RVCAL, RHCAL, defect depth reduction (radiographically)	Untreated periodontitis
Garg & Pradeep [25]	2017	India	RCT	University	90 (30, 30, 30)	90 (30, 30, 30)	A.1. SRP + 1.2% rosuvastatin A.2. SRP + 1.2% atorvastatin B. SRP + placebo	None	Mandibular class II buccal furcations	PD > = 5 mm and horizontal PD > = 3 mm	Single	Single, calibrated, blinded	6 and 9 months	mSBI, PI, PPD, RVCAL, RHCAL, defect depth reduction (radiographically)	Untreated periodontitis
Singhal et al. [26]	2017	India	RCT	University	48 (25, 23)	48 (25, 23)	A. SRP + 0.75% boric acid B. SRP + placebo (Retreatment)	None	Buccal class II furcations of mandibular first molars	PD > = 5 mm and horizontal PD > = 3 mm following SRP	Single, blinded	Single, blinded	3 and 6 months	mSBI, PI, PPD, RVCAL, RHCAL, bone defect depth (radiographically)	Recurrent periodontitis
Pradeep et al. [27]	2013	India	RCT	University	69 (35, 34)	69 (35, 34)	A. SRP + 1% alendronate B. SRP + placebo (Retreatment)	None	Buccal class II furcations of mandibular first molars	PD > = 5 mm and horizontal PD > = 3 mm following SRP	Single	Single, calibrated, blinded	3, 6, 12 months	mSBI, PI, PPD, RVCAL, RHCAL, bone defect depth (radiographically)	Recurrent periodontitis
Pradeep et al. [28]	2012	India	RCT	University	66 (33, 33)	66 (33, 33)	A. SRP + 1.2 mg Simvastatin B. SRP + placebo (Retreatment)	None	Mandibular class II buccal furcations	PD > = 5 mm and horizontal PD > = 3 mm following SRP	Single	Single, calibrated, blinded	3 and 6 months	mSBI, PI, PPD, RVCAL, RHCAL, bone defect depth (radiographically)	Recurrent periodontitis
Tomasi & Wennstrom [29]	2011	Sweden	RCT	University	32 (19, 13)	211 (131, 80)	A. SRP (ultrasonic) + 8.8% doxycycline B. SRP (ultrasonic) only (Retreatment)	Included	First or second molars with grade I to II furcations	PD > = 5 mm or degree I to II furcation involvement	Single, blinded	Two, calibrated, blinded	3 and 9 months	Plaque score, PPD, BOP, location of gingival margin, RAL, degree of horizontal furcation involvement	Recurrent periodontitis
Dannewitz et al. [30]	2009	Germany	RCT	University	39 (19, 20)	Grade I: 41, 60 Grade II: 4, 14 Grade III: 30, 14	A. SRP + 14% doxyxycline B. SRP only (Retreatment)	Included	Furcation grades I, II, III	At least four teeth with residual PD > = 5 mm and BOP. At least one furcation site requiring retreatment	Single, Blinded	Single, blinded	3, 6, 12 months	FMPS, BOP, FMBS, PPD, REC, horizontal and vertical attachment loss	Recurrent periodontitis
Tonetti et al. [31]	1998	Italy	RCT	Private periodontal practices (multicenter)	123 (62, 61)	123 (62, 61)	A. SRP + tetracycline periodontal fibers B. SRP only (Retreatment)	Included	Mandibular class II buccal furcations	At least one mandibular class II furcation with persistent BOP	Different clinicians, blinded	Different examiners, calibrated, blinded	3 and 6 months	BOP, PPD, CAL, REC, plaque score	Recurrent periodontitis

Abbreviations: *BOP* bleeding on probing, *CAL* clinical attachment level, *mSBI* modified sulcus bleeding index, *PI* plaque index, *PPD* probing pocket depth, *RAL* relative attachment level, *RCT* randomized clinical trial, *REC* recession, *RHCAL* relative horizontal clinical attachment level, *RVCAL* relative vertical clinical attachment level, *SRP* scaling and root planing

### Clinical treatment outcome

Clinical data from the included studies are shown in Table 2. All included investigations [24–31] reported data for the clinical treatment outcome. With regards to plaque accumulation, no significant differences were found between the test and control treatment groups in all investigations reporting this variable. Greater improvement in bleeding was reported when alendronate [24], rosuvastatin [25], boric acid [26], simvastatin [28], and tetracycline (only at 3 months) [31] was adjunctively used, compared to SRP only or SRP + placebo. In regard to PPD, greater improvement was noted when SRP was supplemented with alendronate [24, 27], rosuvastatin [25], boric acid [26], simvastatin [28], and tetracycline (only at 3 months), compared to SRP alone or with placebo. With respect to the relative vertical and horizontal clinical attachment level (RVAL and RHAL), a significant benefit was reported after the use of alendronate [24, 27], rosuvastatin [25], boric acid [26], and simvastatin [28] compared to SRP only or placebo. For RHAL, significantly greater improvement was found when doxycycline adjunct to SRP was employed in grade I furcations at 3 months only [30]. In addition, no superiority was found in furcation closure when doxycycline and tetracycline were used [29–31].

**Table 2:** Clinical treatment outcome of the included studies

Study	Plaque	Bleeding	Probing pocket depth (PPD)	Relative vertical clinical attachment level (RVAL)	Relative horizontal clinical attachment level (RHAL)	Additional outcomes
Ipshita et al. [24]	No significant differences at baseline between groups Significant improvement in all groups Baseline: 1.99 ± 0.37 vs 2.01 ± 0.30 vs 2.03 ± 0.40 6 months: 0.76 ± 0.10 vs 0.77 ± 0.09 vs 0.78 ± 0.10 12 months: 0.54 ± 0.11 vs 0.55 ± 0.13 vs 0.57 ± 0.09	No significant differences at baseline between groups Significant improvement in all groups Greater improvement in aloe vera and alendronate groups Baseline: 2.10 ± 0.66 vs 2.06 ± 0.73 vs 2.03 ± 0.61 6 months: 0.70 ± 0.65 vs 0.66 ± 0.60 vs 1.23 ± 0.62 12 months: 0.50 ± 0.50 vs 0.40 ± 0.49 vs 0.90 ± 0.71	No significant differences at baseline between groups Significant improvement in all groups Greater improvement in alendronate group Baseline: 7.03 ± 1.12 vs 7.06 ± 1.32 vs 7.06 ± 1.04 6 months: 4.03 ± 0.55 vs 5.00 ± 0.83 vs 5.53 ± 0.81 12 months: 2.93 ± 0.78 vs 4.63 ± 0.71 vs 5.20 ± 0.66	No significant differences at baseline between groups Significant improvement in all groups Greater improvement in alendronate group Baseline: 6.13 ± 1.00 vs 6.30 ± 0.79 vs 6.23 ± 1.13 6 months: 3.63 ± 1.10 vs 4.26 ± 0.82 vs 5.06 ± 0.78 12 months: 2.36 ± 0.71 vs 3.76 ± 1.35 vs 4.76 ± 0.72	No significant differences at baseline between groups Significant improvement in all groups Greater improvement in alendronate group Baseline: 5.91 ± 0.92 vs 6.16 ± 0.92 vs 6.06 ± 1.08 6 months: 3.56 ± 1.07 vs 4.20 ± 1.20 vs 5.03 ± 0.71 12 months: 2.20 ± 0.61 vs 3.93 ± 1.48 vs 4.60 ± 0.62	None
Garg & Pradeep [25]	No significant differences at baseline between groups Significant improvement in all groups Baseline: 1.87 ± 0.34 vs 1.83 ± 0.30 vs 1.85 ± 0.32 3 months: 0.97 ± 0.25 vs 0.96 ± 0.25 vs 0.99 ± 0.26 6 months: 0.74 ± 0.23 vs 0.73 ± 0.21 vs 0.84 ± 0.28 9 months: 0.55 ± 0.20 vs 0.58 ± 0.24 vs 0.64 ± 0.25	No significant differences at baseline between groups Significant improvement in all groups Greater improvement in rosuvastatin group Baseline: 2.34 ± 0.59 vs 2.29 ± 0.55 vs 2.25 ± 0.58 3 months: 1.01 ± 0.29 vs 1.07 ± 0.32 vs 1.37 ± 0.36 6 months: 0.70 ± 0.17 vs 0.80 ± 0.28 vs 1.20 ± 0.32 9 months: 0.56 ± 0.22 vs 0.65 ± 0.25 vs 1.11 ± 0.28	No significant differences at baseline between groups Significant improvement in all groups Greater improvement in rosuvastatin group Baseline: 7.36 ± 1.12 vs 7.23 ± 1.25 vs 7.63 ± 1.06 3 months: 4.63 ± 0.88 vs 5.26 ± 1.14 vs 6.46 ± 1.69 6 months: 4.06 ± 0.86 vs 4.80 ± 0.92 vs 6.00 ± 1.14 9 months: 3.03 ± 1.18 vs 4.13 ± 0.73 vs 5.80 ± 1.66	No significant differences at baseline between groups Significant improvement in all groups Greater improvement in rosuvastatin group Baseline: 8.56 ± 1.22 vs 8.83 ± 1.11 vs 8.86 ± 1.07 3 months: 5.90 ± 1.24 vs 6.56 ± 1.10 vs 7.56 ± 1.04 6 months: 4.93 ± 1.25 vs 5.83 ± 1.36 vs 7.06 ± 0.86 9 months: 4.06 ± 1.48 vs 4.96 ± 1.12 vs 6.80 ± 0.88	No significant differences at baseline between groups Significant improvement in all groups Greater improvement in rosuvastatin group Baseline: 8.26 ± 1.04 vs 8.43 ± 1.13 vs 8.36 ± 0.99 3 months: 5.96 ± 1.47 vs 6.43 ± 1.25 vs 7.06 ± 1.20 6 months: 5.03 ± 1.32 vs 5.73 ± 1.25 vs 6.50 ± 1.30 9 months: 4.33 ± 1.49 vs 5.10 ± 1.47 vs 6.26 ± 1.25	None
Singhal et al. [26]	No significant differences at baseline between groups Significant improvement in both groups Baseline: 2.01 ± 0.18 vs 1.92 ± 0.17 3 months: 0.86 ± 0.10 vs 1.23 ± 0.15 6 months: 0.46 ± 0.12 vs 0.82 ± 0.15	No significant differences at baseline between groups Greater improvement in boric acid group Baseline: 2.56 ± 0.28 vs 2.53 ± 0.26 3 months: 0.41 ± 0.12 vs 1.44 ± 0.18 6 months: 0.39 ± 0.14 vs 0.73 ± 0.21	No significant differences at baseline between groups Greater improvement in boric acid group Change from baseline to 3 months: 2.20 ± 0.41 vs 1.43 ± 0.51 Change from baseline to 6 months: 3.56 ± 0.51 vs 2.34 ± 0.49	No significant differences at baseline between groups Greater improvement in boric acid group Change from baseline to 3 months: 2.04 ± 0.54 vs 0.95 ± 0.36 Change from baseline to 6 months: 2.92 ± 0.49 vs 1.52 ± 0.51	No significant differences at baseline between groups Greater improvement in boric acid group Change from baseline to 3 months: 1.84 ± 0.47 vs 1.04 ± 0.37 Change from baseline to 6 months: 2.68 ± 0.48 vs 2.13 ± 0.63	None
Pradeep et al. [27]	No significant differences at baseline between groups Significant improvement in both groups Baseline: 1.73 ± 0.31 vs 1.80 ± 0.46 3 months: 0.91 ± 0.27 vs 1.00 ± 0.28 6 months: 0.20 ± 0.40 vs 0.52 ± 0.24 12 months: 0.45 ± 0.19 vs 0.55 ± 0.25	No significant differences at baseline between groups Significant improvement in both groups Baseline: 2.54 ± 0.17 vs 2.53 ± 0.16 3 months: 1.01 ± 0.41 vs 1.15 ± 0.44 6 months: 0.79 ± 0.12 vs 0.88 ± 0.29 12 months: 0.85 ± 0.17 vs 0.90 ± 0.21	No significant differences at baseline between groups Greater improvement in alendronate group Baseline: 6.93 ± 0.69 vs 6.77 ± 0.82 3 months: 4.43 ± 0.73 vs 5.53 ± 0.73 6 months: 3.10 ± 0.71 vs 5.17 ± 0.79 12 months: 3.14 ± 0.71 vs 5.39 ± 0.74	No significant differences at baseline between groups Greater improvement in alendronate group Baseline: 7.30 ± 0.79 vs 7.27 ± 0.78 3 months: 5.07 ± 0.64 vs 6.33 ± 0.66 6 months: 4.07 ± 0.64 vs 6.03 ± 0.76 12 months: 4.07 ± 0.66 vs 6.14 ± 0.89	No significant differences at baseline between groups Greater improvement in alendronate group Baseline: 8.07 ± 0.64 vs 8.03 ± 0.81 3 months: 6.10 ± 0.66 vs 7.17 ± 0.79 6 months: 5.03 ± 0.56 vs 6.97 ± 0.76 12 months: 5.00 ± 0.54 vs 7.03 ± 0.79	None
Pradeep et al. [28]	No significant differences at baseline between groups No significant differences between groups during the study Baseline: 1.88 ± 0.22 vs 1.90 ± 0.20 3 months: 0.97 ± 0.18 vs 0.83 ± 0.15 6 months: 0.68 ± 0.18 vs 0.66 ± 0.18	No significant differences at baseline between groups Significant improvement in both groups Greater improvement in Simvastatin group Baseline: 2.83 ± 0.27 vs 2.51 ± 0.42 3 months: 1.03 ± 0.18 vs 1.42 ± 0.44 6 months: 0.80 ± 0.18 vs 1.61 ± 0.43	Significant improvement in both groups Greater improvement in simvastatin group Baseline: 7.33 ± 1.49 vs 6.80 ± 1.32 3 months: 4.27 ± 0.91 vs 5.13 ± 1.22 6 months: 3.28 ± 0.83 vs 5.50 ± 1.28	Significant improvement in both groups Greater improvement in simvastatin group Baseline: 7.92 ± 1.50 vs 7.43 ± 1.53 3 months: 4.22 ± 1.31 vs 5.07 ± 0.99 6 months: 3.28 ± 1.40 vs 4.97 ± 0.89	Significant improvement in both groups Greater improvement in simvastatin group Baseline: 8.43 ± 1.28 vs 7.86 ± 1.46 3 months: 4.93 ± 0.94 vs 5.56 ± 1.27 6 months: 4.10 ± 1.19 vs 5.43 ± 1.15	None
Tomasi & Wennstrom [29]	No significant differences between groups during the study 3 months: 45 vs 23 6 months: 33 vs 18 12 months: 45 vs 31	No significant differences between groups during the study 3 months: 93 vs 95 6 months: 96 vs 93 12 months: 94 vs 90	No significant differences between groups during the study 3 months: 6.3 vs 6.2 6 months: 5.6 vs 5.7 12 months: 5.7 vs 5.7	No significant differences between groups during the study 6 months: 0.2 vs 0.4 12 months: 0.2 vs 0.3	Not reported	No significant differences in grades I and II furcation closure between groups
Dannewitz et al. [30]	No significant differences at baseline between groups 15.4 ± 7.2 vs 15.7 ± 9.1	No significant differences at baseline between groups 21.2 ± 7.2 vs 24.0 ± 9.9	No significant differences between groups during the study Baseline: 4.96 ± 1.29 vs 4.89 ± 1.01 3 months: 4.03 ± 1.37 vs 4.14 ± 1.12 6 months: 3.85 ± 1.12 vs 3.99 ± 1.04 12 months: 4.08 ± 1.25 vs 4.19 ± 1.18	No significant differences between groups during the study Baseline: 5.96 ± 2.41 vs 5.56 ± 2.20 3 months: 4.88 ± 2.44 vs 4.88 ± 2.30 6 months: 4.56 ± 2.30 vs 4.71 ± 2.16 12 months: 4.69 ± 2.20 vs 4.67 ± 2.12	Significantly greater improvement in doxycycline group at 3 months only (in grade I furcations) Baseline: 5.96 ± 2.41 vs 5.56 ± 2.20 3 months: 4.88 ± 2.44 vs 4.88 ± 2.30 6 months: 4.56 ± 2.30 vs 4.71 ± 2.16 12 months: 4.69 ± 2.20 vs 4.67 ± 2.12	Similar rates of improvement in furcation involvement between groups SRP only led to increase of furcation involvement more often
Tonetti et al. [31]	Not reported	Significant improvement in both groups Greater improvement in tetracycline fibers group at 3 months (not at 6 months) 3 months: 30 vs 48 6 months: 48 vs 48	No significant differences at baseline between groups Greater improvement in tetracycline fibers group at 3 months (not at 6 months) Baseline: 6.3 ± 1.6 vs 5.8 ± 1.7 3 months: 5.0 ± 1.8 vs 5.0 ± 1.5 6 months: 5.0 ± 1.5 vs 4.9 ± 1.4	Not reported	Not reported	No significant clinical attachment loss between groups during the study

### Radiographic treatment outcome and adverse events

Radiographic data from the included studies and adverse events are shown in Table 3. The radiographic bone defect depth and defect depth reduction were reported in five studies [24–28]. Significant improvement was reported in both radiographic outcomes when SRP was supplemented with alendronate [24, 27], rosuvastatin [25], boric acid [26], and simvastatin [28], compared to SRP only or SRP + placebo. Five of the included investigations [24–28] recorded no adverse events, two [29, 30] did not report whether such events occurred during the course of the treatment, while one study reported periodontal abscesses for one control and two test sites [31].

**Table 3:** Radiographic treatment outcome and adverse events of the included studies

Study	Radiographic bone defect depth	Defect depth reduction	Adverse events
Ipshita et al. [24]	No significant differences at baseline between groups Significant improvement in all groups Greater improvement in alendronate group Change from baseline to 6 months: 2.01 ± 0.17 vs 0.76 ± 0.36 vs 0.12 ± 0.04 Change from baseline to 12 months: 2.36 ± 0.31 vs 0.96 ± 0.12 vs 0.19 ± 0.03	Significantly greater in the alendronate and aloe vera groups compared to placebo Greater improvement in alendronate group Change from baseline to 6 months: 38.09 ± 9.53 vs 11.94 ± 15.10 vs 2.12 ± 4.58 Change from baseline 12 months: 44.86 ± 6.29 vs 14.59 ± 25.49 vs 3.77 ± 4.59	None
Garg & Pradeep [25]	No significant differences at baseline between groups Significant improvement in all groups Greater improvement in rosuvastatin group Change from baseline to 6 months: 1.31 ± 0.42 vs 1.06 ± 0.43 vs 0.196 ± 0.15 Change from baseline to 9 months: 1.77 ± 0.39 vs 1.42 ± 0.44 vs 0.213 ± 0.16	No significant differences at baseline between groups Significant improvement in all groups Greater improvement rosuvastatin group Change from baseline to 6 months: 30.80 ± 8.35 vs 25.54 ± 8.89 vs 4.58 ± 3.66 Change from baseline to 9 months: 41.86 ± 6.76 vs 34.31 ± 8.04 vs 4.97 ± 3.62	None
Singhal et al. [26]	Greater improvement in boric acid group Change from baseline to 6 months: 0.68 ± 0.41 vs 0.11 ± 0.03	Greater improvement in boric acid group. Change from baseline to 6 months: 16.98 ± 1.03 vs 2.86 ± 0.92	None
Pradeep et al. [27]	No significant differences at baseline between groups Greater improvement in alendronate group Change from baseline to 6 months: 1.27 ± 0.28 vs 0.11 ± 0.003 Change from baseline to 12 months: 1.29 ± 0.27 vs 0.07 ± 0.06	Greater improvement in alendronate group Change from baseline to 6 months: 32.11 ± 6.18 vs 2.71 ± 0.61 Change from baseline to 12 months: 32.66 ± 5.86 vs 1.83 ± 1.51	None
Pradeep et al. [28]	No significant improvement in placebo Significant improvement in simvastatin group Change from baseline to 6 months: 1.15 ± 0.61 vs 0.06 ± 0.25	Greater improvement in simvastatin group At 6 months: 25.16 vs 1.54	None
Tomasi & Wennstrom [29]	Not reported	Not reported	Not reported
Dannewitz et al. [30]	Not reported	Not reported	Not reported
Tonetti et al. [31]	Not reported	Not reported	One control and two test sites developed periodontal abscess

The studies’ conclusions are demonstrated in Table 4. Overall, SRP in conjunction with alendronate, rosuvastatin, boric acid, and simvastatin led to significantly better treatment outcomes compared to SRP alone or with placebo [24–28]. Doxycycline showed only short-term superior effects in one study [30], whereas similar outcomes were demonstrated in another study [29]. Tetracycline fibers resulted in superior periodontal outcomes only short-term [31].

**Table 4:** Studies’ conclusions based on their findings

Study	Conclusions
Ipshita et al. [24]	Alendronate showed significant improvement in all clinical parameters, along with greater defect depth reduction, compared to aloe vera in the treatment of class II furcation defects as an adjunct to SRP
Garg & Pradeep [25]	Rosuvastatin showed significant improvement in all clinical parameters along with significantly greater defect depth reduction as compared to atorvastatin group in treatment of mandibular class II furcation defects as an adjunct to SRP
Singhal et al. [26]	Boric acid showed significant improvement in clinical parameters compared to placebo gel as an adjunct to SRP
Pradeep et al. [27]	Local delivery of 1% alendronate into a class II furcation defect stimulates a significant PD reduction, RVCAL and RHCAL gains, and improved bone fill compared with placebo gel as an adjunct to SRP
Pradeep et al. [28]	Locally delivered simvastatin provides a comfortable and flexible method to improve clinical parameters and also to enhance bone formation
Tomasi & Wennstrom [29]	Improvement in molar furcation involvement after non-surgical periodontal therapy was not enhanced by adjunctive locally applied doxycycline
Dannewitz et al. [30]	Single subgingival application of doxycycline in addition to SRP had a short-term effect on furcation involvement
Tonetti et al. [31]	Application of tetracycline fibers in addition to SRP had a short-term effect on periodontal parameters during periodontal maintenance of class II mandibular furcations

### Risk of bias

**Fig. 2:**
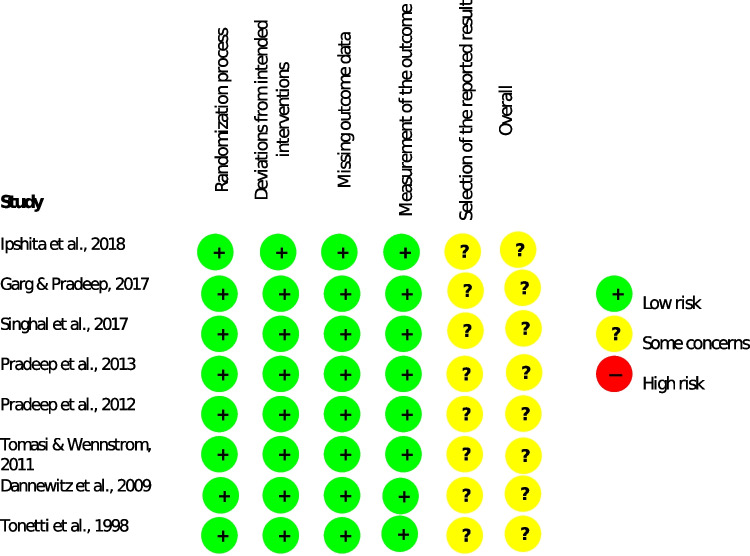
Risk of bias assessment of the included studies

The risk of bias assessment of the included studies is presented in Fig. 2. All publications were assessed according to the revised tool for risk of bias assessment of randomized clinical trials using ROB 2.0 (Fig. 2). All included studies demonstrated “some concerns” with respect to the selection of the reported results, and therefore the overall risk of bias outcome was “some concerns” [24–31].

## Discussion

The need for additional periodontal treatment, either non-surgical or surgical, following the initial periodontal treatment is often observed in periodontitis cases. This is more frequent especially in molars with furcation defects [15]. Adjunctive treatments have been utilized to overcome this limitation of non-surgical periodontal therapy with the aim to eliminate the need for periodontal surgery. This may be primarily important for medically compromised periodontitis patients who are on several medications. Nevertheless, this is important even for medically healthy patients when the risk of gingival recession is imminent as it occurs commonly after periodontal flap surgical procedures [32]. To our knowledge, there is no systematic review assessing the effect of local delivery products in furcation defects. Therefore, the aim of the present systematic review was to evaluate the effect of subgingival administration of various drugs and host-modulating agents in furcation defects, as adjuncts to non-surgical periodontal therapy, compared to non-surgical periodontal treatment alone or combined with placebo.

Findings from this systematic review showed that the majority of the available publications depicted beneficial treatment outcomes, both clinically and radiographically, following the use of local adjuncts to non-surgical periodontal therapy (SRP) in furcation defects. Superiority was primarily noted for PPD reduction and RHAL and RVAL gain as well as bone defect depth reduction. Notably, due to the variability in the agents used in each study and the methodological differences between studies using the same agent, a meta-analysis was not possible. Specifically, alendronate was tested in two RCTs [24, 27], one of which included the use of the host-modulating agent at the initial active periodontal therapy [24], whereas the other study utilized it during the retreatment/reinstrumentation stage [27]. With regards to doxycycline, the two included studies utilized the medication in different concentrations, as well as in different furcation defects [29, 30].

Non-surgical periodontal treatment is considered the gold standard in periodontal therapy, but subgingival instrumentation may still lead to residual pockets [15]. Residual pockets of at least 5 mm should be retreated non-surgically, while periodontal surgeries should be employed in sites with at least 6 mm PPD [33]. The recently introduced S3 clinical guidance further introduces the third step of periodontal therapy consisting of selective retreatment of residual active sites following the application of subgingival debridement [15]. Thus, it becomes important to eliminate the occurrence of residual deep pockets following the initial phase of periodontal therapy in order to avoid additional periodontal surgeries. Various adjuncts to non-surgical periodontal therapy have been utilized including physical or chemical agents (laser, photodynamic therapy), host-modulating agents (local or systemic) such as statins, probiotics, bisphosphonates, non-steroidal antiinflammatory drugs, fatty acids, and metformin, as well as subgingival locally delivered antimicrobials and systemic antimicrobials [15]. The use of local therapies is advantageous because of their ability to deliver therapeutic levels of the agents directly into the periodontal pockets with no systemic adverse effects and no or minor local adverse effects [34]. Especially for furcation defects, the use of local therapies is of paramount importance.

With regards to the host-modulating agents, this review identified studies reporting on the use of alendronate and statins, such as rosuvastatin, atorvastatin, and simvastatin. Specifically, the local application of alendronate in mandibular grade II furcation defects demonstrated significantly enhanced clinical and radiographic treatment outcomes, when used in conjunction with SRP, compared to SRP with placebo in step II and step III periodontal treatment [24, 27]. Alendronate is an amino bisphosphonate inhibiting bone resorption and leading to increased bone mineral density, osteoblast induction, and bone deposition [35–37]. Topical application of alendronate has shown beneficial effects when used during the initial periodontal treatment [37, 38]. In addition, alendronate has been utilized in intrabony defects during periodontal surgery, leading to superior pocket depth reduction, attachment level gain, and periodontal bone repair when compared to placebo [39].

Moreover, statins including rosuvastatin, atorvastatin, and simvastatin have been tested for their additional effect as adjunctive treatment to SRP for the treatment of mandibular grade II furcation defects and have shown significantly improved clinical and radiographic treatment outcomes, compared to SRP + placebo [25, 28]. Their adjunctive effect in periodontal therapy is primarily a result of their antiinflammatory and antimicrobial properties, as well as because of the improved wound healing associated with their use [40]. Moreover, statins inhibit osteoclast differentiation and improve the production of bone anabolic factors enhancing osteoblastic differentiation and bone formation [41]. A network meta-analysis aiming to assess the effect of locally delivered statins for the treatment of chronic periodontitis in combination with SRP demonstrated a significant superiority, even in type 2 diabetes and smoker patients [42]. Rosuvastatin led to significantly greater intrabony defect bone fill, compared to atorvastatin and simvastatin [42]. Finally, another meta-analysis demonstrated additional significant improvements for PPD reduction and CAL gain when statins were used locally as adjuncts to SRP, with atorvastatin and rosuvastatin leading to the greatest improvement [43].

With regard to antiseptics, this review identified a study reporting on the adjunct use of boric acid. Boric acid has demonstrated antimicrobial and antiinflammatory properties by inhibiting the lipopolysaccharide-induced tumor necrosis factor-α (TNF-α) [44, 45]. In addition, it reduces bone resorption by inhibiting osteoclastogenesis and increases osteogenic effects by stimulating osteogenic differentiation and osteoblastogenesis [46]. Furthermore, systemic administration of boric acid has been shown to reduce alveolar bone loss in a diabetic rat model [44]. A recent meta-analysis concluded that the adjunctive use of boric acid in non-surgical periodontal therapy results in improved treatment outcomes at 3 and 6 months, with no adverse events [47]. The present systematic review included a study evaluating the efficacy of 0.75% boric acid gel as an adjunct to SRP for the treatment of buccal grade II furcations of mandibular first molars [26]. Greater improvement for inflammation, PPD, RVAL, RHAL, radiographic bone defect depth, and defect depth reduction was reported when the boric acid gel was applied in furcation defects, compared to placebo, during reinstrumentation [26].

Finally, with regard to antibiotics, this review identified studies reporting on doxycycline and tetracycline fibers. Doxycycline is a third-generation tetracycline leading to better absorption, protein binding, and diffusion into the tissues and exhibiting prolonged action, when compared to tetracycline [48]. Furthermore, doxycycline is characterized by antimicrobial properties and is an inhibitor of enzymes involved in connective tissue breakdown [49]. Local application of doxycycline gel as an adjunct to non-surgical periodontal therapy has shown positive outcomes in periodontitis patients [50] and leads to improved clinical attachment in smokers [51]. In the present review, the adjunctive use of local doxycycline in furcation defects did not show any significant superiority compared to SRP alone [29, 30] or demonstrated only short-term benefits in grade I furcations [30]. Similarly, short-term improvement was only reported for deep periodontal pockets in a multicenter 12-month study that recruited subjects with recurrent or persistent periodontitis during supportive periodontal treatment [52]. On the other hand, tetracycline fibers have also been associated with significant clinical benefits when combined with SRP in a number of studies [53, 54]. However, this reported clinical superiority has been supported by short-term clinical studies of 3–6 months duration [53, 54]. In agreement with these findings, furcation defects treated with tetracycline fibers showed greater inflammation and pocket depth reduction at 3 months compared to SRP alone [29]; however, this effect did not remain at 6 months following the treatment [31].

To conclude, according to the present systematic review, host-modulating agents including alendronate, statins, and antiseptic such as boric acid may lead to notable improvement after non-surgical periodontal treatment of grade II furcation defects. However, it is important to consider the small number of included studies and the different therapeutic products and protocols assessed. Specifically, two studies utilized these agents during the initial periodontal treatment [24, 25], whereas the rest of the included studies incorporated the agents during the maintenance phase [26–31]. Moreover, all investigations presented some concerns about the risk of bias assessment due to the lack of information regarding the reported outcome selection. Furthermore, another risk of bias should be considered. Five of the totally eight included clinical trials [24–28] were conducted in the same country (India), which may introduce geographical bias, and therefore additional data from heterogeneous populations would be needed to draw meaningful and universal conclusions. The site was the unit of analysis for locally delivered medications/agents in furcation defects (topical application). Most of the included studies considered only one site per patient. However, few studies considered more than one site with the same characteristics (furcation defect) per patient. This is considered a limitation of the present systematic review. Another limitation worth noting is that the vast majority of agents discussed in the present review are currently not approved for use by the US Food and Drug Administration (FDA) or are not available in a number of countries; therefore, the clinical relevance of their adjunct use is disputable. Overall, the scarce research data on the effect of local adjunctive treatment in furcation defects reveals the definite need for further well-designed RCTs adopting standardized protocols to evaluate the long-term clinical and radiographic treatment outcomes following such interventions.

## Conclusion

In conclusion, based on the currently available evidence derived from RCTs, local antimicrobial and host-modulating agents including alendronate, rosuvastatin, boric acid, simvastatin, and tetracycline may enhance the clinical treatment outcomes when used as adjuncts to non-surgical periodontal treatment in furcation defects. Host modulators including alendronate, rosuvastatin, and simvastatin as well as boric acid demonstrated more favorable radiographic treatment outcomes than conventional periodontal therapy in molars with furcation defects. All agents appeared to be overall safe and well-tolerated and were not associated with adverse reactions. Future and adequately powered studies are needed to confirm their long-term effectiveness including different populations and further explore the effects of these agents on challenging clinical scenarios.

## Data Availability

Data available on request from the authors.
